# Time-Dependent Risk for Recurrence in Survivors of Major Adverse Cardiovascular Events

**DOI:** 10.7759/cureus.59366

**Published:** 2024-04-30

**Authors:** Anderson Bermon, Belem Trejo-Valdivia, Carlos Federico Molina Castaño, Angela M Segura, Norma C Serrano

**Affiliations:** 1 Centro de Investigaciones, Fundación Cardiovascular de Colombia, Bucaramanga, COL; 2 Escuela de graduados, Universidad CES, Medellín, COL; 3 Centro de Investigación en Nutrición y Salud, Instituto Nacional de Salud Pública, Cuernavaca, MEX; 4 Epidemiology, Tecnológico de Antioquia Institución Universitaria, Medellin, COL

**Keywords:** acute myocardial infarc, cardiovascular prevention, cox proportional hazards regression, prospective cohort, major adverse cardiovascular event, survival analysis

## Abstract

Introduction: The prevalence of the population with a history of an occlusive cardiovascular event has been increasing in recent years, which means that a large number of patients will have a higher risk of presenting a fatal recurrence. The aim is to determine variables associated with time-to-recurrent cardiovascular events and analyze how changes in low-density lipoprotein cholesterol (LDL-C) levels during follow-up may be associated with this time-to-event.

Materials and methods: This is a prospective observational cohort study of 727 adults with a history of at least one occlusive cardiovascular event recruited at a referral hospital in northeastern Colombia. Data from a follow-up period of a maximum of 33 months (median 26 months) (one death) were used to define how clinical and sociodemographic variables impact the recurrence of major adverse cardiovascular events (MACE). Analyses were performed based on proportional hazard models and time-dependent hazard models.

Results: Upon enrollment, 215 (30%) of the participants reported experiencing their most recent cardiovascular event within the preceding year. After two years, the recurrence rate was 12.38% (90/727). The risk of recurrence before two years was 3.9% (95% CI 2.7-5.6). In the multiple models, the presence of severe depression gives a Hazard Ratio of 8.25 (95% CI 2.98-22.86) and LDL ≥120 md/dl Hazard Ratio of 2.12 (95% CI 1.2 -3.9). It was found that LDL >120 mg/dl maintained over time increases the chances of recurrence by 1.7% (Hazard Ratio: 1.017, 95% CI 0.008-0.025).

Conclusions: The present study allows us to identify a profile of patients who should be treated promptly in an interdisciplinary manner to avoid recurrences of coronary events.

## Introduction

The remarkable improvement in survival rates following major cardiovascular events, ranging from 89% to 97%, coupled with global population aging, has significantly contributed to the nearly doubled prevalence of total cardiovascular disease cases (CVDs) [1-3]. An analysis of available data reveals this substantial increase in the global burden of these diseases between 1990 and 2019, the number of individuals affected rose from an estimated 271 million (95% uncertainty interval: 257 to 285 million) to 523 million (95% uncertainty interval: 497 to 550 million) [4]. This significant rise has cemented CVDs as the leading cause of global morbidity [4].

While significant progress has been made in identifying factors associated with CVD in middle-income countries [5], information on factors contributing to event recurrence remains limited. This is particularly crucial as this growing population faces a fourfold higher risk of mortality attributed to potential recurrent cardiovascular events compared to the general population [6,7]. Reported recurrence rates at one and three years range from 3.5 to 19 per 100 individuals [8], with mortality varying between 3.1 and 24.7%, depending on individual comorbidities [9,10].

To address this critical knowledge gap, the present study aims to determine variables associated with time-to-recurrent cardiovascular events and analyze how changes in low-density lipoprotein cholesterol (LDL-C) levels during follow-up may be associated with this time-to-event. This comprehensive approach will not only identify factors that influence recurrent event risk but also shed light on the dynamic interplay between LDL-C management and time to recurrence.

## Materials and methods

Study design and data sources

Follow-up contact was made with all patients enrolled in the clinical study “Txt2Heart Colombia” a prospective, observational, and pragmatic cohort study involving patients treated at a high-complexity center, a reference in northeastern Colombia. The patients had a history of being diagnosed with acute coronary syndrome (unstable angina, acute myocardial infarction with and without ST elevation), stable angina, ischemic cerebrovascular events, and peripheral obstructive arterial disease [11]. The follow-up period extended for a maximum duration of 33 months, encompassing the years 2017 to 2020, with the primary objective of assessing recurrent major adverse cardiovascular events (MACE). 

**Figure 1:**
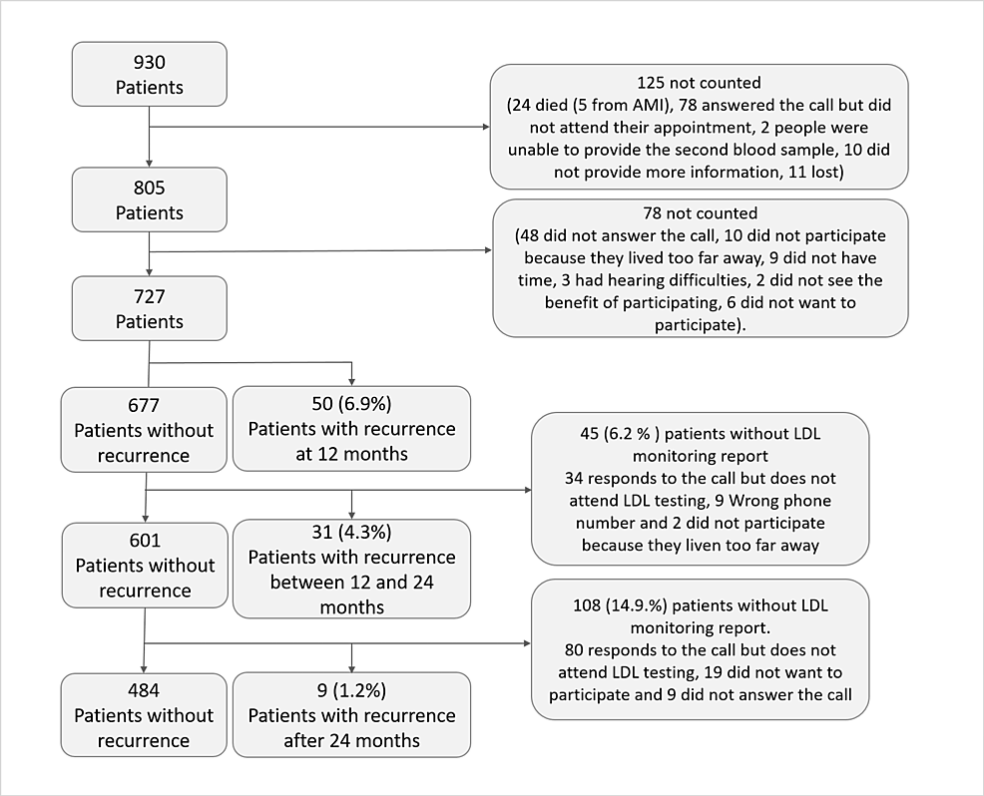
Participant selection flow chart AMI: Acute Myocardial Infarction

Among the 930 initially recruited patients, 727 (78.2%) were included in the current analysis. Out of the excluded patients, 125 could not be reached for the second year, and 78 had only one follow-up measure despite being approached in the first year, leading to their exclusion from the analysis. Figure 1 provides further details regarding the reasons for non-contact.

At the time of recruitment, a total of 215 patients (29.6%) had experienced a coronary event in the previous year, while 388 (53.3%) and 124 (17.1%) had experienced such events between one and five years and more than five years before, respectively. Having identified the date of the last cardiovascular event prior to recruitment made it possible to evaluate the recurrence time as the period between this and the date of recurrence reported during the follow-up period; thus, the shortest reported follow-up time was one month, and the greater than 207 months since the last event.

Tracking and defining events

From 2017 to 2020, patients underwent a minimum of three face-to-face encounters with the study’s medical staff. The first contact served as the baseline measurement, the second occurred at a 12-month interval (within a 15-day window), and the third was conducted after 24 months (within a 30-day window) of the baseline measurement. The interviews included questions regarding event occurrences, hospitalizations, and interventions. A general practitioner from the research group collected blood pressure, weight, and height measurements every three months during follow-ups, utilizing the same calibrated instruments. The information was recorded in specific questionnaires in CommCare (www.commcarehq.org) and REDCap (www.project-redcap.org) [12]. A copy of the medical records that reported recurrences or death certificates was obtained with consent from the patients or their relatives.

Definition of MACEs

The outcome variable evaluated was the recurrence of major adverse cardiovascular events (MACEs), defined as death from cardiovascular causes, hospitalization due to a cardiovascular event, the necessity for revascularization (percutaneous, bypass), stroke, or acute myocardial infarction. These events were confirmed by clinical history and assessed by the general practitioner. In all cases, confirmation was required by a note in the medical records by the treating cardiologist. In instances of uncertainty, confirmation was sought from the cardiologist within the research group upon reviewing the medical record or relevant paraclinical records, such as angiographies and echocardiograms. MACEs were considered a recurrence when symptoms appeared 28 days after the resolution of the previous event, without the involved organ needing to be the same as the last event or events.

MACEs Predictors

Non-modifiable variables were sex, age, and primary basic education. Modifiable variables included risk of depression as identified by the Patient Health Questionnaire-9 (PHQ-9), low-density cholesterol (LDL), ultra-sensitive C-reactive protein (CRP), systolic blood pressure (SBP), diastolic blood pressure (DBP), pulse, body mass index (BMI), work status and the use of medications such as antihypertensives, beta-blockers, antiplatelet agents and acetylsalicylic acid (ASA). The non-modifiable variables were collected only once at the beginning of the study. Among the modifiable variables, the PHQ-9, ultra-sensitive CRP, and smoking had only one measurement at first contact. The remaining variables had at least two measurements.

For the statistical analyses, quantitative variables were categorized based on levels reported in the literature as a risk for CAD. The PHQ-9, with values <5, was considered "no risk of depression", values from 5 to 14 were categorized as "medium/moderate risk" and values ≥15 were considered "risk of severe depression"[13]. Age was categorized as less than 60 and ≥60 years since the last cardiovascular event presented at the baseline measurement. The level of schooling was categorized with a cutoff of five years, and patients with five or more years of study were labeled as "with primary basic education"[14]. This variable was considered non-modifiable, as even if the patient pursued studies during the follow-up period, socio-cultural conditions inherent to the studies were estimated to influence the risk of CVD over time [15,16]. Employment status was categorized as “currently working,” “retired,” and “unemployed.” Smoking history was classified as having never smoked, stopped smoking more than one year ago, and active smoker. The BMI was categorized as “normal” (18.5-24.9 kg/m^2^), “overweight” (25.0-29.9 Kg/m^2^), and “obesity” (≥ 30 Kg/m^2^) [17].

Different LDL cut-off points were evaluated, including 50 mg/dl, 70 mg/dl, 100 mg/dl, and 120 mg/dl [18-20], considering multiple recommendations from the literature. Heart rate was considered less than 90 beats per minute, systolic blood pressure (SBP) of 160, and diastolic blood pressure (DBP) of 90. The cut-off point for Ultrasensitive C-reactive protein (CRP) was 2 µ/ml as an inflammatory risk marker for a heart attack [21]. Medications included in the study were those with well-demonstrated effectiveness in preventing MACEs, recommended by various clinical guidelines and consensuses [6,22]. Registration of these medications was based on their function or type of therapeutic action, resulting in five groups: “antihypertensives”, “beta-blockers”, “antiplatelet agents”, “statins” and “ASA” [23].

Statistical analysis

An exploratory analysis of baseline characteristics, including sociodemographic variables, cardiovascular risk factors, and clinical and treatment data, was carried out. Some continuous variables were categorized based on clinical parameters. Associations between MACE and each of the variables were determined to select those as potentials to be included in a Cox proportional hazard model (CPHM), for the construction of the final multiple model, inclusion of variables that are statistically significant in the simple regression models was conducted. These preliminary models aim to explore which variables may be influencing the explanation of the dependent variable. Additionally, variables with biological plausibility or those reported in the literature are included [24]. Possible interactions between variables were evaluated using linear regression models.

Crude recurrence rates of MACEs were calculated based on the Kaplan-Meier estimator. Additionally, complementary double logarithmic plots for each covariate were used as entry criteria to the Cox model. Multiple CPHM was adjusted using baseline variables, and a Cox model with time-dependent variables [25] was considered to evaluate the effect on the risk of recurrence of those variables changing along the follow-up (such as DBP, LDL, working condition and without taking antihypertensives). In both cases, the point estimate and 95% confidence interval for hazard ratios (HR) were reported. The statistical diagnosis of Cox models was based on the analysis of Martingale, Cox-Snell, and Schoenfeld residuals. Statistical analyses were performed using STATA version 17.0 (StataCorp LLC., College Station, TX).

Ethics approval

The study was granted ethical approval by the Comité de Ética en Investigaciones de la Fundación Cardiovascular de Colombia (CEI_506-013330). The investigators adhered rigorously to the established ethical standards for scientific research at both national and international levels throughout the research process. This study was partially funded by Universidad CES (Medellín, Colombia), and grants 844-2019 (code 656684467847) and 753-2016 from MINCIENCIAS (Colombia's Ministry of Science, Technology and Innovation) and the authors declare no conflicts of interest.

## Results

Prospective follow-up was carried out on 727 patients with a history of obstructive cardiovascular events (atherosclerotic cardiovascular disease, ASCVD) from April 2017 to March 2020. The baseline characteristics of the patients are described in Table 1.

**Table 1:** Baseline characteristics of the included patients AMI: Acute Myocardial Infarction, PAD: Peripheral Arterial Disease, LDL: Colesterol Low-Density Lipoproteins, SBP: Systolic Blood Pressure, DBP: Diastolic Blood Pressure, BMI: Body Mass Index, “normal” (18.5-24.9 kg/m^2^), “overweight” (25.0-29.9 Kg/m^2^), and “obesity” (≥ 30 Kg/m^2^). *There are only 492 patients with ultra-sensitive C-reactive protein (CRP)

Risk factor	Censored n (%)	MACEs n (%)	p
Unmodifiable			
Sex			0.877
Male	500 (87.7%)	70 (12.3%)	
Female	137 (87.3%)	20 (12.7%)	
Age at last event			0.992
>60 years	333 (87.6%)	47 (12.4%)	
≤ 60 years	304 (87.6%)	43 (12.4%)	
Education			0.092
Without basic education	252 (85.1%)	44 (14.9%)	
With basic education	385 (89.3%)	46 (10.7%)	
Previous cardiovascular event			0.04
AMI	550 (87.6%)	78 (12.4%)	
Stable angina	42 (97.7%)	1 (2.3%)	
Stroke	25 (83.3%)	5 (16.7%)	
PAD	20 (76.9%)	6 (23.1%)	
Modifiable			
PHQ-9 (depression)			0.006
Risk free	471 (89.5%)	55 (10.5%)	
Medium/moderate risk	156 (83.9%)	30 (16.1%)	
Severe Depression	10 (66.7%)	5 (33.3%)	
LDL basal			0.67
>120 mg/dl	519 (87.4%)	75 (12.6%)	
≤ 120 mg/dl	118 (88.7%)	15 (11.3%)	
SBP >160			0.046
>160 (mmHg)	41 (78.8%)	11 (21.2%)	
≤ 160 (mmHg)	596 (88.3%)	79 (11.7%)	
DBP >90			0.558
>90 (mmHg)	33 (84.6%)	6 (15.4%)	
≤ 90 (mmHg)	604 (87.8%)	84 (12.2%)	
Heart rate (bpm)			0.494
>80 (bpm)	89 (85.6%)	15 (14.4%)	
≤ 80 (bpm)	548 (88%)	75 (12%)	
Ultrasensitive CRP*			0.972
>2 µ/ml	10 (83.3%)	2 (16.7%)	
≤ 2 µ/ml	415 (86.5%)	65 (13.5%)	
BMI			0.641
Normal	161 (87.5%)	23 (12.5%)	
Overweight	306 (86.7%)	47 (13.3%)	
Obesity	170 (89.5%)	20 (10.5%)	
Working condition			<0.001
Currently working	271 (93.4%)	19 (6.6%)	
Retired	248 (84.6%)	45 (15.4%)	
Jobless	118 (81.9%)	26 (18.1%)	
Smoking			0.337
Has never smoked	16 (80%)	4 (20%)	
Ex-smoker	245 (86.3%)	39 (13.7%)	
Active smoker	376 (88.9%)	47 (11.1%)	
Diabetes mellitus			0.341
Yes	86 (90.5%)	9 (9.5%)	
No	545 (87.1%)	81 (12.9%)	
Antihypertensives			0.319
Yes	430 (88.5%)	56 (11.5%)	
No	207 (85.9%)	34 (14.1%)	
Statins			0.799
Yes	575 (87.5%)	82 (12.5%)	
No	62 (88.6%)	8 (11.4%)	
Beta blockers			0.177
Yes	532 (88.4%)	70 (11.6%)	
No	105 (84%)	20 (16%)	
Antiplatelet agents			0.034
Yes	561 (86.7%)	86 (13.3%)	
No	76 (95%)	4 (5%)	
Acetylsalicylic acid			0.451
Yes	569 (87.9%)	78 (12.1%)	
No	68 (85%)	12 (15%)	

The total number of recurrences was 90 (12%). There was a total of 47,098 days of follow-up; the patient with the longest contribution time was 208 months, and the one with the shortest time was 1.6 months. The probability of presenting a recurrence at two years was 3.9% (95% CI 2.7-5.6), and at five years was 10.5% (95% CI 8.2-13.3). The results of the Simple Proportional Composite Risk Model, multiple Proportional Composite Risk Model, and Time Dependent Model are presented in Table 2.

**Table 2:** Cox models for MACEs recurrences MACE: Major Adverse Cardiovascular Events, AMI: Acute Myocardial Infarction, PAD: Peripheral Arterial Disease, LDL: Low-Density Lipoproteins, SBP: Systolic Blood Pressure, DBP: Diastolic Blood Pressure, BMI: Body Mass Index, “normal” (18.5-24.9 kg/m^2^), “overweight” (25.0-29.9 Kg/m^2^), and “obesity” (≥ 30 Kg/m^2^). *There are only 492 patients with ultra-sensitive C-reactive protein (CRP).

Predictor	Simple model Hazard Ratio (CI 95%)	Multiple model Hazard Ratio (CI 95%)	Time dependent model Hazard Ratio (CI 95%)
Gender (Male)	1.09 (0.66-1.79)	1.50 (0.83-2.70)	1.57 (0.88-2.83)
Age (>60 years)	1.13 (0.75-1.72)	-	-
Without basic education	1.39 (0.92-2.11)	1.52 (0.97-2.38)	1.43 (0.91-2.26)
PHQ-9 (depression)			
Risk free	1	1	1
Medium/moderate risk	1.59 (1.02-2.47)	1.84 (1.16-2.92)	1.85 (1.16-2.92)
Severe Depression	4.56 (1.82-11.43)	8.25 (2.98-22.86)	8.21 (2.97-22.67)
Basal LDL >120 mg/dl	1.53 (0.87-2.67)	2.12 (1.16-3.88)	3.76 (1.92-7.36)
Basal LDL >100 mg/dl	1.32 (0.84-2.10)	-	-
Basal LDL >70 mg/dl	1.46 (0.95-2.23)	-	-
Basal LDL >50 mg/dl	1.36 (0.55-3.39)	-	-
SBP >160 mmHg	1.36 (0.86-2.13)	-	-
DBP >70 mmHg	1.27 (0.83-1.94)	1.52 (0.99-2.35)	1.45 (0.95-2.24)
Pulse >80 bpm	1.01 (0.66-1.55)	-	-
Ultrasensitive CRP (>2 µ/ml)	1.26 (0.77-2.05)	-	-
BMI		-	-
Normal	1	-	-
Overweight	1.23 (0.74-2.07)	-	-
Obesity	1.12 (0.62-2.02)	-	-
Working condition			
Currently working	1	1	
Retired	2.05 (1.20-3.51)	2.25 (1.29-3.92)	2.36 (1.34-4.14)
Jobless	2.43 (1.35-4.42)	2.88 (1.52-5.45)	2.87 (1.50-5.49)
Smoking			
Has never smoked	1	-	-
Ex smoker	0.74 (0.49-1.14)	-	-
Active smoker	1.31 (0.47-3.66)	-	-
Diabetes mellitus	1.50 (0.95-2.37)	-	-
Without Antihypertensives	1.34 (0.87-2.06)	1.71 (1.09-2.66)	1.67 (1.07-2.60)
Statins	1.21 (0.58-2.51)	-	-
Beta blockers	0.85 (0.52-1.41)	-	-
Acetylsalicylic acid	0.98 (0.54-1.81)	-	-
LDL monitoring >120 mg/dl	N/A	N/A	1.017 (1.008-1.025)

In the simple analysis, LDL >100 mg/dl and LDL >50 mg/dl were excluded due to a lack of statistical significance, such as smoking status, diabetes mellitus, beta-blocker use, and ASA. Although DBP <70 mmHg presented a p-value >0.2, its clinical relevance led to its inclusion in the Multiple model, which showed significance (Table 2).

**Figure 2:**
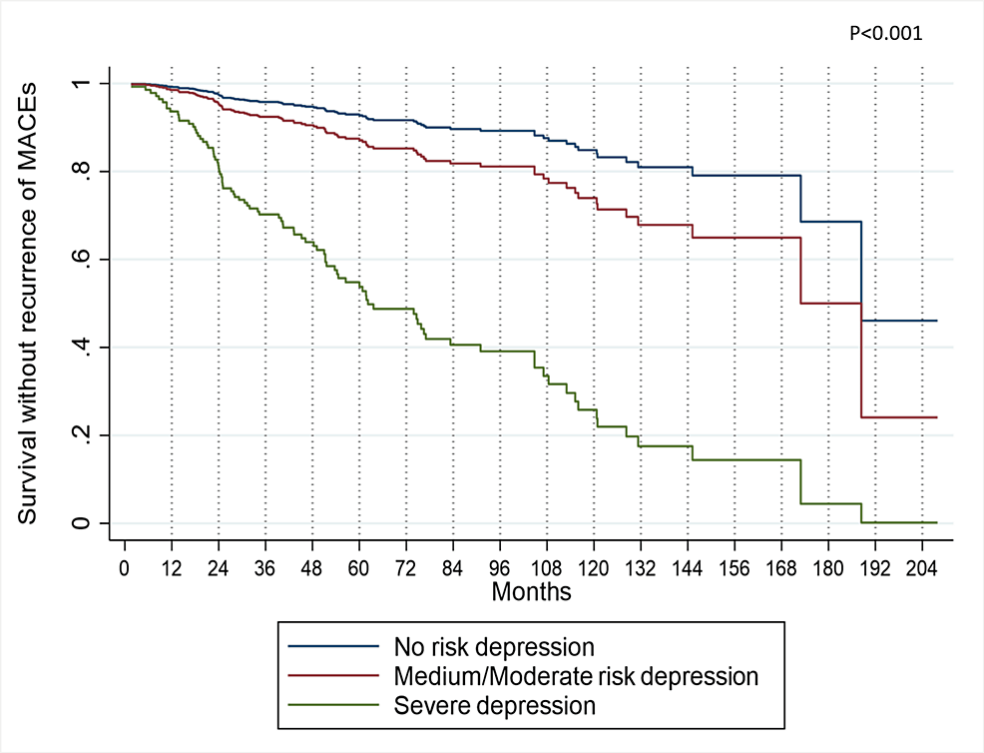
MACEs recurrence-free survival, according to PHQ-9, adjusted by the variables of the multiple Cox model MACE: Major Adverse Cardiovascular Events

In the Cox proportional hazard model, we assessed all possible two-way interactions between the model's variables and sex, employment status (with PHQ-9, age, and education), and DBP (with antihypertensives). No statistically significant interactions were identified. By employing the Cox Proportional Hazards regression model and utilizing the mean value of each variable, the specific impact of PHQ-9 on the recurrence risk of MACEs was investigated. Results reveal that after two years, individuals with severe depression exhibit a 20% likelihood of experiencing some form of recurrence. In contrast, patients categorized as having medium to moderate depression risk and those with no depression risk display probabilities of MACE recurrence at 5% and 3%, respectively. Additionally, the probability of recurrence reaches 45% for patients with severe depression within a five-year timeframe, contrasting with the rates of 12.5% and 7.5% observed for those with medium to moderate risk and no risk of depression, respectively (Figure 2).

**Figure 3:**
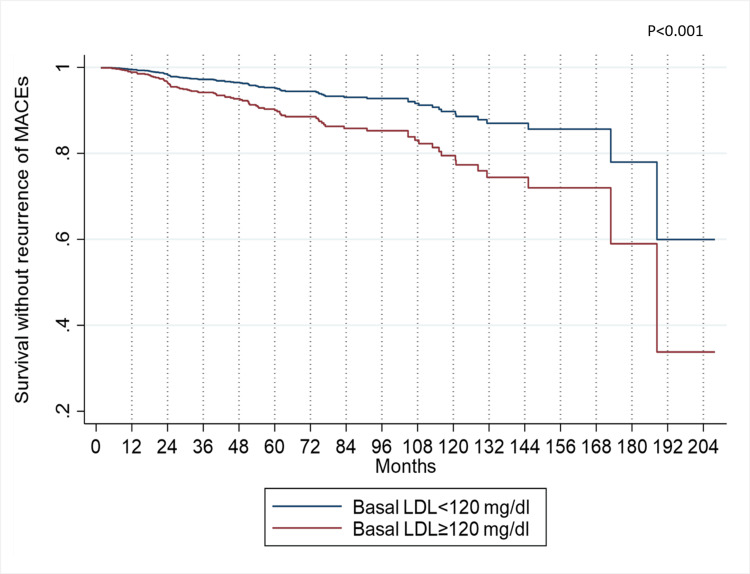
MACEs recurrence-free survival, according to LDL adjusted by the variables of the multiple Cox model MACE: Major Adverse Cardiovascular Events, LDL: Low-Density Lipoproteins.

The five-year probability of recurrence-free survival, stratified by baseline LDL levels exceeding 120 mg/dl, stood at 90%. In contrast, patients with basal LDL levels below this threshold exhibited a higher probability of 95%. This distinction becomes more pronounced over 10 years, with recurrence-free survival probabilities of 79% for individuals surpassing the LDL threshold and 90% for those below it (Figure 3).

Time dependent model

Dependent variables studied were LDL levels >120 mg/dl, the utilization of antihypertensives, and systolic blood pressure >70 mmHg. Initial exploration showed that LDL was the only variable contributing to the model. The first day of contact was marked as the beginning of follow-up, which was marked as zero time point (t0). Blood samples were taken to assess LDL levels, and systolic blood pressure was also recorded at that moment.

Follow-up assessments were scheduled at 12 ± 1 months, 24 ± 2 months, and 30 ± 4 months post-zero time point. Notably, 727 (100%) patients completed LDL follow-up at one year, 45 (6.2%) patients did not provide the LDL value for follow-up at 24 months, and 108 (14.9%) at 30 months. Loss to follow-up was attributed to several factors. A portion of participants declined to return for blood draws to assess LDL-C levels. Others withdrew from further study participation, and in other cases, attempts to reach participants by phone for follow-up were unsuccessful due to either inactive phone lines or incorrect contact information. The primary event of interest for analysis was the initial recurrence of MACEs post-zero time point. Subsequent recurrences were not factored into the analysis despite continued patient monitoring.

In the time-dependent model, severe depression remains the variable with the highest risk, showing an HR of 8.21 (95% CI 2.97-22.67). The study sample shows a trend regarding the significance of the variables from the previous model. While the follow-up LDL presents an HR of 1.017 (95% CI 1.008-1.025), its descriptive significance level and narrow confidence interval indicate its precision and relevance. It is observed that changes in LDL levels influence the probability of recurrence over time. The lowest probability of recurrence is found in patients whose LDL remained below 120 mg/dL in both baseline and follow-up measurements. Following them are patients who initially had LDL levels below 120 mg/dL but later had levels equal to or greater than 120 mg/dL during follow-up. In third place are patients whose baseline LDL measurements were equal to or greater than 120 mg/dL but decreased to values below 120 mg/dL during follow-up. Finally, the group at highest risk consists of patients whose LDL remained equal to or greater than 120 mg/dL throughout baseline and follow-up measurements.

**Figure 4:**
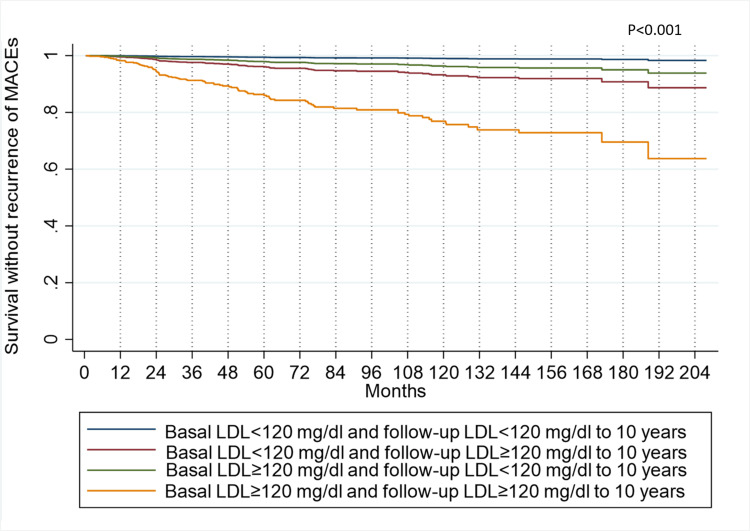
MACEs recurrence-free survival, according to the best-case scenario and LDL follow-up at 10 years of the multiple Cox model with time-dependent covariates MACE: Major Adverse Cardiovascular Events, LDL: Low-Density Lipoproteins

The aforementioned trends are depicted in Figure 4, illustrating the Model variables adjusted to the best-case scenario and LDL follow-up at 10 years. In this, patients with LDL levels ≥120 mg/dL who cannot change them have a 1.6% probability of experiencing a recurrence within the first year.

**Figure 5:**
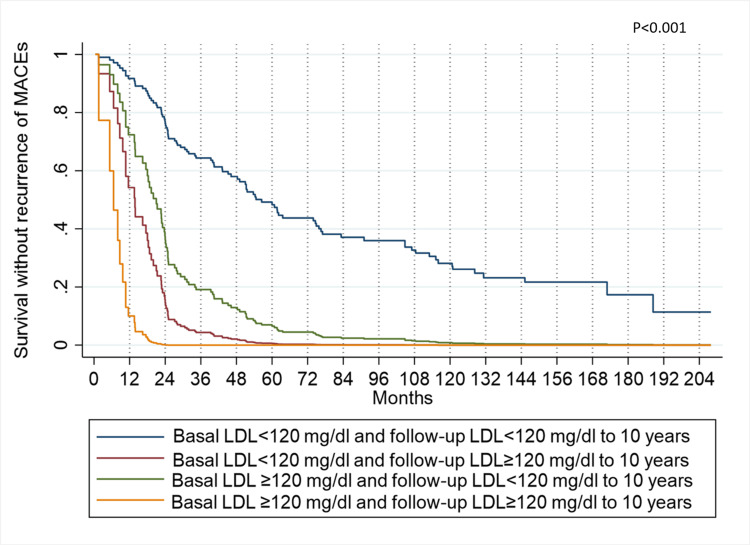
MACEs recurrence-free survival, according to the worst-case scenario and LDL follow-up at 10 years of the multiple Cox model with time-dependent covariates MACE: Major Adverse Cardiovascular Events, LDL: Low-Density Lipoproteins.

On the other hand, Figure 5 displays the Model variables adjusted to the worst-case scenario and LDL follow-up simultaneously. Here, patients with LDL levels ≥120 mg/dL who cannot change them face a 90% probability of recurrence within the first year.

**Figure 6:**
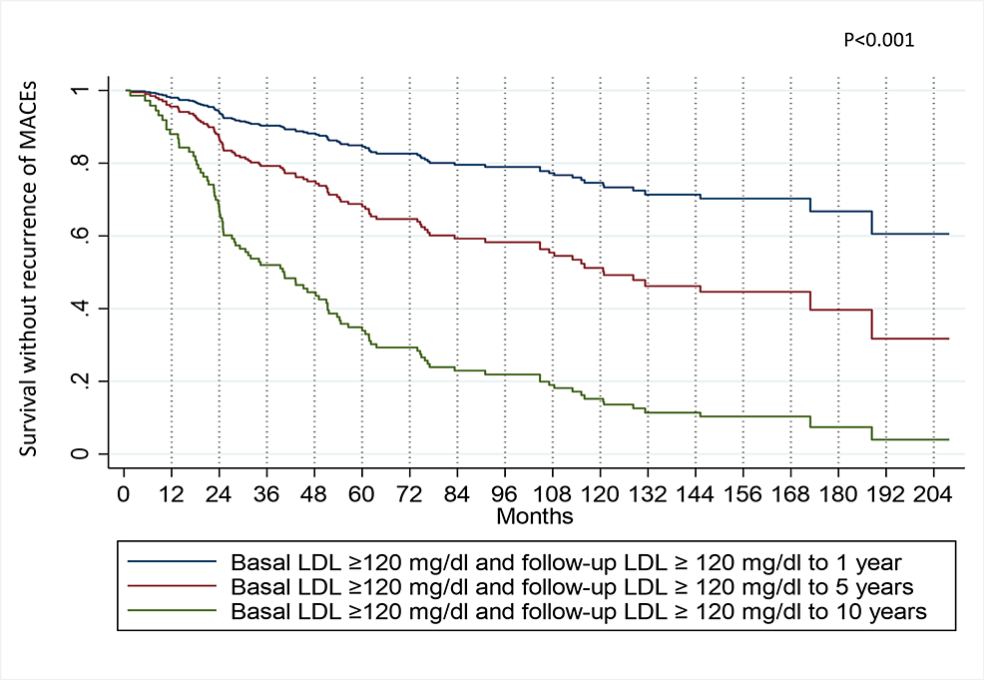
MACEs recurrence-free survival in patients who do not modify their high LDL levels during one, five, and 10 years The patients were examined using the multiple Cox model with time-dependent covariates. MACE: Major Adverse Cardiovascular Events, LDL: Low-Density Lipoproteins.

Figure 6 illustrates how the longer it takes to lower LDL levels, the higher the recurrence probability. Each variable in the model is adjusted to its average, considering the persistence of LDL at values equal to or greater than 120 mg/dL. A discernible trend emerges wherein the probability of remaining recurrence-free progressively diminishes as high LDL levels persist over time. Specifically, this probability stands at 94% at two years for patients whose baseline and follow-up LDL levels persisted at or above 120 mg/dl over one year, subsequently decreasing to 86% at the same two-year mark for those whose LDL levels did not change over five years of follow-up. For patients who failed to reduce their LDL levels during the 10-year follow-up period, the probability drops to 65% at the two-year mark.

**Figure 7:**
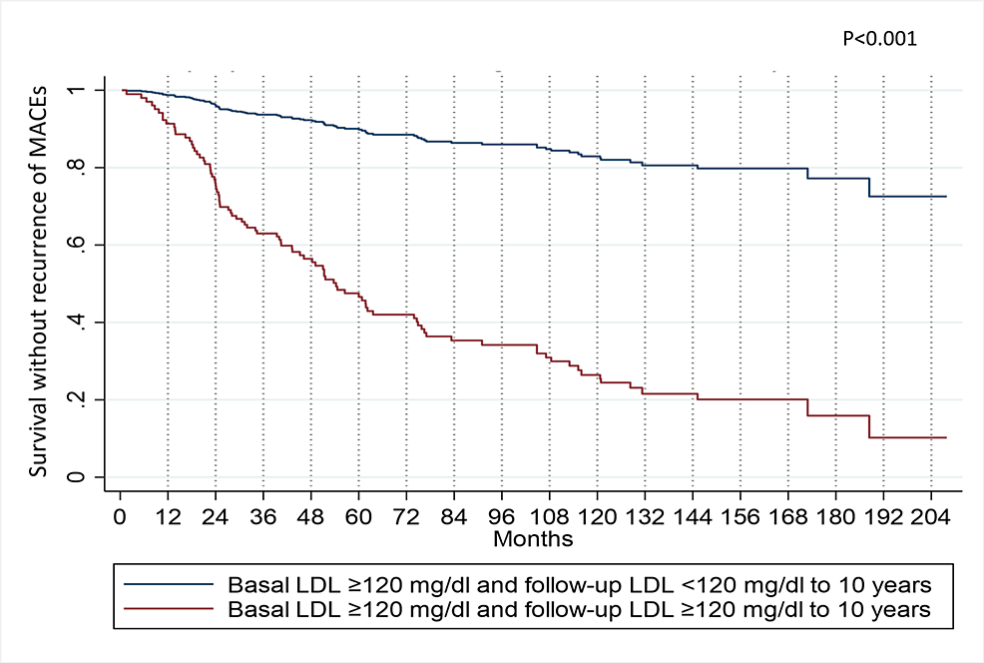
MACEs recurrence-free survival in patients who successfully modify their LDL levels during a 10-year follow-up period The patients were assessed using the multiple Cox model with time-dependent covariates. MACE: Major Adverse Cardiovascular Events, LDL: Low-Density Lipoproteins.

Figure 7 demonstrates a notable surge in the probability of recurrence-free survival from MACEs when patients successfully lower their LDL levels over a 10-year follow-up period. Specifically, at the five-year mark, the likelihood of recurrence for patients maintaining LDL levels ≥120 mg/dL stands at 45%. In contrast, for those who achieve LDL reduction to values <120 mg/dL during the same timeframe, it diminishes to 8%. These findings are derived from model adjustments based on variable averages. This implies that reducing LDL levels during follow-up can enhance the likelihood of remaining free from recurrence. Nevertheless, this probability will never surpass that of patients whose baseline LDL measurements were below 120 mg/dL.

## Discussion

The findings of the present study replicate and enhance the conclusions of previous research, establishing the association between depression and MACEs, even when adjusted for age and sex. It was possible to demonstrate how the presence of a high risk of depression, identified by the PHQ-9 scores, can influence the speed of appearance of MACEs even when adjusted for sociodemographic and clinical variables. A similar study in the United States, after a 12-month follow-up of patients with a history of a coronary event, showed an increased risk associated with moderate-to-severe self-reported depressive symptoms, with an HR=1.72 (95% CI = 1.02-2.89) even in covariate-adjusted models [26]. The present study has demonstrated an HR of 8.25 (95% CI 2.98-22.86), higher than those reported in other works.

The prevalence and persistence of depression in patients with acute myocardial infarction have been reported between 7.3 and 31.1% [27], with evidence that somatic affective depressive symptoms are more strongly associated with mortality and cardiovascular events in patients with heart disease (HR 1.19, 95% CI 1.10-1.29) [28]. There is still controversy about the most efficient treatment for these patients, although pharmaceutical interventions have reported good results [29].

The LDL variable has been associated with the increased risk of MACEs and their rate of occurrence. The present study reports an HR of 2.12 in the multiple model, the only variable that showed increased risk over time. A Spanish cohort presented results indicating a higher risk of recurrence in patients with lower LDL compared to patients who experienced a MACE for the first time (110.12 mg/dl vs. 131.6 mg/dl, p<0.01, respectively) [30]. However, despite analyzing this variable across multiple strata, only the threshold level of 120 mg/dL was found to be significant in the models.

LDL reduction has become an important goal in preventing atherosclerotic cardiovascular disease due to its association with atherosclerosis [22,31]. It has been observed that patients experience a decrease in LDL after the first coronary event. However, after the first year of follow-up, fewer achieve the goals established by the clinical practice guidelines due to inadequate follow-up (lipid-lowering titration) or low adherence to statins. This highlights the need to intensify long-term interventions to reach LDL cholesterol goals and maximize secondary prevention [32]. While there are recommendations to reduce LDL to values below 55 mg/dl, the present study demonstrated a significant difference starting from 120 mg/dl. This does not challenge the established parameter but instead emphasizes an anticipated effect over time for a specific population. Simultaneously, the absence of pharmacological antihypertensive treatment occurred in 33.15% of the population. This is considered an incomplete therapeutic approach to secondary prevention of patients with a history of coronary events.

From a sociodemographic perspective, the trend between the lack of primary education and the employment situation with the speed of occurrence of events is striking. Notara et al., in a 10-year follow-up study, demonstrated that patients with a history of coronary syndrome with less than nine years of schooling (low level of education) had twice the risk of all-cause mortality compared to those with nine years to 14 years of education (medium level of schooling) and more than 14 years of education (high level of schooling) (relative frequency of 40% versus 22% and 19% respectively, p<0.001) [33]. However, an effect on this risk was observed when adjusting several factors for situation, including financial situation, better nutrition, work stress, among others. Although it is a challenge to determine the impact of modifying the educational level in a population similar to that of this study, the importance of developing secondary prevention strategies that promote equitable scenarios, which encompass access to healthy lifestyles and coverage of needs basic in the short and long term, it is emphasized.

Regarding employment status, studies have demonstrated that patients with more severe coronary events and higher comorbidity exhibit lower rates of return and access to employment, which may be a predictive variable rather than a causal one in the presence of recurrence [34]. It is essential to clarify that the interaction between the employment status variable and the patient's age was ruled out.

For time-dependent variables, a history of acute myocardial infarction and LDL have been identified as factors that increase the risk of death with an HR of 2.05 (95% CI 1.39-3.02; p<0.0001) and HR of 1.06 (95% CI 1.02-1.11, p 0.007), respectively, at five years [35]. These findings align with the present study, although no coefficients had been previously presented demonstrating the yearly increase in risk. While the current study does not aim to establish a conclusive association between LDL and the presence of MACEs [35-37], it underscores the importance of timely intervention over time and the impact that achieving secondary prevention control goals early can have. This is particularly crucial in the health system of a developing country where access limitations, non-adherence to treatment guidelines, and delays in follow-up can contribute to the recurrence of MACEs.

Strengths and limitations

This is the first study that includes time-dependent variables in an expanded Cox model to determine how time influences the probability of experiencing a recurrence of a cardiovascular event. Added strength of the study was the hospital center where the cohort was monitored, which is a reference institution in northeastern Colombia, has a high volume of patients and an acute myocardial infarction center of excellence certified by the Joint Commission International, which guarantees the quality of patient monitoring and treatment.

Although the study´s main finding was the association of depression with the recurrence of events, only the baseline measurement of this condition was available. This limitation prevented the adjustment of the model based on the evolution of depression over time. However, within its predictive and non-causal concept, the model allows the identification and prompt intervention of this condition. Another limitation was entering the study cohort sometime after the last event, with an average of 26 months. However, the authors consider that this a common scenario in clinical practice; this factor complicates the determination of exposure time for various conditions and establishes an association of the variables with a temporality directly linked to the last event.

## Conclusions

The present study enables the identification of a population profile that should be promptly treated in an interdisciplinary manner to prevent recurrent coronary events. This poses a challenge for health services to identify high-risk patients and provide effective care to achieve goals and address conditions such as depression in a timely and effective manner, ultimately leading to a tangible decrease in recurrences in the short term. 

## Appendices

Data are available on reasonable request.
